# Rapports sexuels chez les élèves de la commune de Matoto à Conakry en Guinée

**DOI:** 10.11604/pamj.2020.35.113.20733

**Published:** 2020-04-13

**Authors:** Abdourahamane Diallo, Yaya Diallo, Aboubacar Sidiki Magassouba, Ibrahima Koussy Bah, Telly Sy

**Affiliations:** 1 Service de Gynécologie-Obstétrique, Hôpital National Ignace Deen, CHU de Conakry, Conakry, Guinée; 2 Faculté des Sciences et Techniques de la Santé, Université Gamal Abdel Nasser de Conakry, Conakry, Guinée

**Keywords:** Rapports sexuels, élèves adolescents, Matoto

## Abstract

**Introduction:**

L'objectif de ce travail était d'étudier les facteurs associés à la pratique des rapports sexuels chez les élèves adolescents de la commune de Matoto à Conakry.

**Méthodes:**

Il s'agissait d'une étude transversale, descriptive et analytique étendue sur 3 mois (1^er^ mars - 31 mai 2018) ayant concerné les élèves âgés de 10 à 19 ans fréquentant les collèges et lycées de la commune de Matoto à Conakry.

**Résultats:**

Parmi les 500 élèves interrogés, 226 (162 filles et 64 garçons) déclarent avoir eu des rapports sexuels soit une fréquence de 45,2%. Le préservatif n'était utilisé que par 16,4% des élèves et les contraceptifs par 35,4%. La fréquence des infections sexuellement transmissibles était de 23,5%. Parmi les filles ayant eu des rapports sexuels, 32,1% ont eu au moins une grossesse et celle-ci s'est soldée par un avortement clandestin dans 30,8% des cas. Le facteur associé à la réalisation des rapports sexuels chez les adolescents était les sorties nocturnes (p = 0,000).

**Conclusion:**

Les rapports sexuels sont fréquents chez les élèves adolescents de la commune de Matoto. Le préservatif et les contraceptifs sont peu utilisés. Il ressort aussi de notre étude que les sorties nocturnes étaient le facteur le plus associé à la pratique.

## Abstract (en)

**Introduction:**

The purpose of this study is to analyze the factors associated with sexual practices among adolescent students in Matoto, Conakry.

**Methods:**

We conducted a cross-sectional, descriptive and analytical study of students aged 10-19 years attending colleges and high schools in Matoto, Conakry over a period of 3 months (1 March -31 May 2018).

**Results:**

Out of 500 students surveyed, 226 (162 girls and 64 boys; 45.2%) reported having sexual intercourse. Condom was used by 16.4% of students and contraceptives by 35.4%. The rate of sexually transmitted infections was 23.5%. Among girls who had sexual intercourse, 32.1% had had at least one unintended pregnancy ending in clandestine abortion in 30.8% of cases. The factor associated with sexual practices among adolescents was night life (p = 0.000).

**Conclusion:**

Sexual intercourse is frequent among adolescent students in Matoto. Condom and contraceptives are little used. In our study, night life was the principal factor associated with sexual practice.

## Introduction

L'adolescence se caractérise par la survenue des transformations physiques, biologiques et psychologiques, lesquelles entrainent des changements profonds notamment sur le comportement sexuel des individus [1]. Cette période coïncide au début de l'activité sexuelle chez plusieurs adolescents. En France, la proportion d'individus âgés de 15 à 19 ans sexuellement actifs est de 7,2% chez les garçons et 5,9% chez les filles alors qu'elle est respectivement au Cameroun de 11,5% et 18%, à Madagascar de 8,4% et 17,2%, au Rwanda de 15,3% et 5,2%, en Côte d'ivoire de 16,7% et 20,4% [2]. Selon une étude menée dans les écoles de la ville de Likasi en République Démocratique du Congo, 42% des élèves sont sexuellement actifs, 38% ont eu leurs premiers rapports sexuels entre 10 et 15 ans et 56% déclarent avoir eu plus d'un partenaire sexuel [3]. Ce comportement sexuel est influencé par les medias, l'internet, le niveau et le milieu de vie des jeunes [4]. En Guinée, 17,9% des garçons et 19,7% des filles âgés de 15 à 19 ans sont sexuellement actifs soit l'un des taux les plus élevés en Afrique [2]. Cette initiation sexuelle précoce expose les adolescents à un risque accru d'infection par les IST/VIH, de grossesses non désirées, d'avortements à risque et d'abandon scolaire. En outre, la santé des jeunes filles est compromise car elles sont plus sujettes à la morbidité et à la mortalité maternelle en raison de leur immaturité physiologique [5-7]. Mabiala Babela *et al.* rapportent qu'en cas de grossesse non désirée, une interruption volontaire de grossesse a été pratiquée dans 64,7% des cas et le taux d'abandon scolaire enregistré à l'occasion d'une grossesse s'élevait à 82,4% [1]. L'importance du retentissement potentiel des rapports sexuels sur la vie et la santé actuelles et futures des adolescents et la rareté d'études sur cette question en Guinée ont motivé à la réalisation de ce travail qui avait pour objectif d'étudier les facteurs associés à la pratique des rapports sexuels chez les élèves adolescents de la commune de Matoto à Conakry.

## Méthodes

Il s'agissait d'une étude transversale à visée descriptive et analytique qui s'est déroulée sur une période de 3 mois allant du 1^er^ mars au 31 mai 2018 qui a concerné les élèves âgés de 10 à 19 ans fréquentant les collèges et lycées de la commune de Matoto à Conakry en Guinée. N'ont pas été inclus tous les élèves mariés et ont été exclus tous les élèves non mariés qui n'ont pas accepté de participer à l'étude. Pour déterminer le nombre d'élèves à inclure dans l'étude, la formule suivante a été utilisée :

n = (z)^2^ p (1 - p) / d^2^

n = taille de l'échantillon ; z = niveau de confiance selon la loi normale centrée réduite (pour un niveau de confiance de 95%, z = 1.96) ; p = proportion estimée de la population qui présente la caractéristique (en se basant sur une enquête préliminaire réalisée dans la commune de Kaloum, la proportion d'élèves ayant eu des rapports sexuels est de 50%). d = marge d'erreur tolérée (par exemple on veut connaître la proportion réelle à 5% près). Les calculs ont donné un effectif de 384 élèves qui a été majoré à 500 élèves. Nous avons décidé volontairement d'interroger 10 élèves par école ce qui correspond à 50 écoles pour avoir l'échantillon de 500.

A cause de l'effectif important d'établissements secondaires et d'élèves dans cette commune, il a été procédé à un échantillonnage aléatoire à deux degrés. Au premier degré, par un échantillonnage aléatoire simple, il a été sélectionné 50 collèges et lycées (publiques et privés, enseignement général et franco-arabe) dans lesquels l'étude a eu lieu. Au second degré, les élèves de ces écoles ont été répartis en deux groupes suivant le sexe (filles et garçons). Par la suite, il a été recruté de façon aléatoire simple parmi les filles, 7 élèves (à cause de leur plus grande vulnérabilité) et parmi les garçons 3 élèves soit au total 10 élèves dans chacun des collèges et lycées retenus. Cela a permis d'obtenir un échantillon de 500 élèves composé de 350 filles et 150 garçons qui ont fait l'objet d'une collecte de données à l'aide d'un formulaire d'enquête préalablement établi et testé. Les données ont été collectées par interview directe des élèves par l'enquêtrice qui a été formée à cet effet. Les variables étudiées étaient les suivantes : l'âge des élèves, le type d'établissement, le niveau d'instruction, les rapports sexuels, l'âge aux premiers rapports sexuels, les motifs des rapports sexuels, l'utilisation des préservatifs, la multiplicité des partenaires, la contraction d'une IST, la réalisation d'un test de VIH, le résultat du test de VIH, la survenue d'une grossesse, l'utilisation des contraceptifs, l'excision et le comportement à risque. Les données collectées ont été saisies avec Excel 2013 et analysées avec le logiciel SPSS version 16.

Sur le plan descriptif, nous avons calculé des proportions avec leurs intervalles de confiance à 95% pour toutes les variables. Les facteurs associés à la pratique des rapports sexuels ont été recherchés par une analyse bivariée et multivariée. Au cours de l'analyse bivariée, la comparaison de proportions a été faite par le test de Khi2 de Pearson lorsque tous les effectifs théoriques étaient plus grands que 5, par le Kh2 corrigé de Yates lorsqu'au moins un des effectifs théoriques est inférieur à 5 mais plus grand que 2,5 et par le test exact de Fisher lorsqu'au moins un des effectifs théoriques est inférieur à 2,5. L'analyse multivariée a été faite par régression logistique avec comme variable à expliquer la pratique des rapports sexuels par les élèves adolescents. Pour le choix des variables explicatives à introduire dans le modèle de régression, la procédure de pas à pas descendante a été appliquée.

Les résultats sont donnés en p-value, odds-ratio brute (ORb) et odds-ratio ajusté (ORa) avec les intervalles de confiance à 95% correspondant à chaque variable explicative. L'association a été jugée significative lorsque p est inférieur à 5% ou lorsque l'intervalle de confiance des ORb et ORa exclut la valeur 1.

**Ethique :** sur le plan éthique, l'accord préalable du comité national d'éthique, du directeur communal de l'éducation de Matoto, des proviseurs des lycées et des principaux des collèges a été obtenu avant le recrutement des élèves. Le consentement éclairé des élèves a été obtenu avant la collecte des données. La confidentialité et l'anonymat ont été respectés.

## Résultats

**Attitudes des élèves adolescents vis-à-vis des rapports sexuels** (Tableau 1): près de la moitié des élèves ont déjà eu au moins une fois les rapports sexuels et près de neuf élèves sur dix ayant eu des rapports sexuels l'ont eu entre 15 et 19 ans et sans contrainte dans 77% des cas.

**Tableau 1:** Répartition des élèves adolescents de la commune de Matoto en fonction de leur attitude vis-à-vis des rapports sexuels

Variables	Effectifs	Pourcentage	IC à 95%
**Avez-vous eu des rapports sexuels (n=500)**			
Oui	226	45,2	[40,6-49,6]
Non	274	54,8	[50,4-59,4]
**Age des premiers rapports sexuels (n=226)**			
Moins de 15 ans	30	13,3	[8,8-17,7]
15 à 19 ans	196	86,7	[82,3-91,2]
**Circonstance des rapports sexuels (n=226)**			
Amour	79	35	[30,7-39,3]
Contrainte	52	23	[19,4-26,6]
Argent	44	19,5	[14,8-24,2]
Curiosité	51	22,6	[12,3-32,9]

**Attitude des élèves adolescents vis-à-vis du VIH et des IST** (Tableau 2)**:** parmi les 226 élèves ayant eu des rapports sexuels, près de la moitié des élèves (43,8%) ont eu 2 ou plusieurs partenaires sexuels et seulement 37 (16,4%) ont utilisé le préservatif. L'utilisation du préservatif a été systématique chez 27 élèves sur les 37 qui l'ont utilisé. La fréquence des IST était de 23,5% et le test de VIH a été réalisé par 5,8% des élèves ayant commencé les rapports sexuels et résultat était positif pour un seul élève.

**Tableau 2:** Répartition des 226 élèves adolescents des collèges et des lycées de Matoto ayant eu des rapports sexuels en fonction de leur attitude vis-à-vis du VIH et des IST

Variables	Effectifs	Pourcentage	IC à 95%
**Nombre de partenaires sexuels (n=226)**			
1	127	56,2	[51,5-61,4]
2 ou plus	99	43,8	[39,6-48,5]
**Utilisation du préservatif (n=226)**			
Oui	37	16,4	[11,5-21,2]
Non	189	83,6	[78,8-88,5]
**Utilisation systématique du préservatif (n=37)**			
Oui	27	73	[59,5-86,5]
Non	10	27	[13,5-40,5]
**Avez-vous eu une IST (n=226)**			
Oui	53	23,5	[18,1-29,2]
Non	173	76,5	[70,8-81,9]
**Avez-vous réalisé un test de VIH (n=226)**			
Oui	13	5,8	[3,1-8,8]
Non	213	94,2	[91,2-96,9]
**Quel était le résultat (n=13)**			
Positif	1	7,7	[2,2-12,5]
Négatif	12	92,3	[87,5-97,8]

**Attitudes des élèves adolescents vis-à-vis de la grossesse** (Tableau 3): la majorité des élèves ayant eu des rapports sexuels n'ont jamais utilisé une méthode contraceptive (64,6%) et 32% des filles qui ont eu des rapports sexuels ont contracté une grossesse. Cette grossesse n'était pas désirée dans 92,3% des cas, s'est soldé par un avortement clandestin dans 30,8% des cas.

**Tableau 3:** Répartition des élèves adolescents des collèges et des lycées de Matoto ayant eu des rapports sexuels en fonction de leur attitude vis-à-vis de la grossesse

Variables	Effectif	Pourcentage	IC à 95%
**Utilisez-vous un contraceptif (n=226)**			
Oui	80	35,4	[28,6-42,2]
Non	146	64,6	[57,8-71,4]
**Avez-vous eu une grossesse (n=162)**			
Oui	52	32,1	[25,3-39,2]
Non	110	67,9	[60,8-74,7]
**Aviez-vous désiré cette grossesse (n=52)**			
Oui	48	92,3	[87,8-97,2]
Non	4	7,7	[2,8-12,2]
**Issue de la grossesse (n=52)**			
Accouchement	27	51,9	[38,5-65,4]
Avortement clandestin	16	30,8	[19,2-42,3]
Avortement spontané	9	17,3	[7,7-28,8]

**Facteurs associés aux rapports sexuels** (Tableau 4): les facteurs qui ont été significativement associés à la réalisation des rapports sexuels sont les sorties nocturnes, l'usage de la drogue, l'usage du tabac, l'usage de l'alcool avec une p-value de 0,000 pour tous ces facteurs.

**Tableau 4:** Répartition 226 élèves adolescents des collèges et des lycées de la commune de Matoto selon le sexe, le type d’établissement, la sortie nocturne, l’usage du tabac, de l’alcool et de la drogue

Variable	Rapport sexuel		P-value	OR brute	IC 95%
	Oui (n=226)	Non (n=274)			
**Sexe**					
Masculin	64 (42,0%)	87 (58,0%)	0,347	0,8	[0,6-1,2]
Féminin	162 (46,6%)	187 (53,4%)			
**Type d'établissement**					
Privé	177 (44,4%)	222 (55,6%)	0,454	0,8	[0,5-1,3]
Publique	49 (48,5%)	52 (51,5%)			
**Sortie nocturne**					
Oui	199 (85,0%)	36 (15,0 %)	0,000	50,1	[29,3-85,7]
Non	27 (10,2 %)	238 (89,8 %)			
**Usage du Tabac**					
Oui	34 (94,4%)	2 (5,6%)	0,000	24,1	[5,7-101,4]
Non	192 (41,4%)	272 (58,6%)			
**Usage de l’alcool**					
Oui	43(100%)	0(0%)	0,000	2,5	[2,5-2,8]
Non	183 (40%)	274 (60%)			
**Usage de la drogue**					
Oui	21 (95,5%)	1(4,5%)	0,000	28	[3,7-209,6]
Non	205 (42,9%)	273 (57,1%)			

**Régression logistique** (Tableau 5)**:** après la prise en compte de l'interaction entre les différents facteurs étudiés, ce sont les sorties nocturnes qui sont associées à la réalisation des rapports sexuels avec une p-value de 0,000.

**Tableau 5:** Analyse multivariée par régression logistique

Variables	OR ajusté	IC_95%_	p-value
Tabagisme	3,58	[0,1-1,1]	0,166
Alcool	5,00	[0,5-24,2]	0,985
Sortie nocturne	40,14	[22,7-70,9]	0,000

## Discussion

Les rapports sexuels sont fréquents et précoces chez les élèves de la commune de Matoto. Près d’un élève adolescent sur deux a eu déjà un rapport sexuel (45,2% [40,6-49,6]) et dans 13% des cas avant même l’âge de 15 ans. Cette fréquence est supérieure à celle rapportée par Godeau *et al.* en 2008 (17,7%) [8] mais est identique à celle de Mabiala Babela à Brazzaville (50%) [1]. La virginité chez la fille qui était considérée pendant le mariage dans les sociétés africaines comme un signe de bonne éducation et qui amenait les filles à éviter les rapports sexuels avant d'être mariées perd de plus en plus son poids avec les nouvelles générations. Cela peut s'expliquer par l'accès facile même pour les plus jeunes à des vidéos pornographiques que l'on connait actuellement à la télévision ou à l'internet qui contribue à créer l'envie d'avoir les rapports sexuels chez les adolescents et à les conduire vers l'accomplissement précoce de cet acte [4]. Verdure *et al.* ont rapporté que 40% d'adolescents de sexe masculin trouvent intéressant de parler de la pornographie au cours des séances d'éducation sexuelle [9]. Selon Puglia et Bulot, les adolescents consommateurs de pornographie soulignent davantage les effets positifs associés à la pornographie, ils reconnaissent une influence de ce média sur leur vie sexuelle et ils adoptent des pratiques sexuelles plus diversifiées que celles de leurs pairs non-consommateurs [4, 10]. Par contre, pour Smaniotto *et al.* il n'y a pas de consensus au tour de l'influence de la pornographie sur la sexualité adolescente [11]. Le nombre d’élève ayant eu des rapports sexuels consentis par amour (35% [30,7-39,3]) ou par curiosité (22,6% [12,3-32,9]) au cours de cette étude traduit l’envie d’avoir des rapports sexuels chez ces élèves. Ce même constat a été fait par Pettifor *et al.* qui, dans leur étude sur la précocité des premiers rapports sexuels en Afrique du Sud, parlent de 96 à 97% des jeunes garçons et 57 à 61% de jeunes filles qui disent avoir participé volontairement à leurs premiers rapports sexuels et 83 à 84% des hommes et 27 à 32% des femmes déclarent avoir « vraiment » désiré leurs premiers rapports sexuels [7]. En revanche, Adriana au Ghana et Decraen au Rwanda ont rapporté respectivement que 58% et 15,5% de jeunes ont été forcés à avoir des rapports sexuels [12, 13]. La pauvreté contribue également à entretenir cette sexualité précoce car 19,5% [14,8-24,2] des élèves ayant eu des rapports sexuels les ont eus à cause de l’argent. Talnan *et al.* ont rapporté dans leur étude menée en Côte d'Ivoire que l'entrée en vie sexuelle se fait de manière plus précoce dans les couches sociales défavorisées et la susceptibilité d'avoir des rapports sexuels avec plusieurs partenaires est plus élevée pour ces jeunes que pour ceux vivant dans des conditions socio-économiques meilleures [14].

La fréquence élevée des rapports sexuels chez cette couche vulnérable de la population à cause de son immaturité et de son inexpérience entraine des multiples conséquences à court et à long terme (IST/VIH, grossesses non désirées, avortement provoqué, accouchement prématuré, infertilité, cancer du col utérin, décès maternel et infantile, échec et/ou abandon scolaire etc.) [7, 12]. Cette situation est aggravée par la faible utilisation du préservatif (16,4% [11,5-21,2]) et des méthodes contraceptives (35,4% [28,6-42,2]) ainsi que la multiplicité des partenaires chez 43,8% d’élèves âgés de 10 à 19 ans. Dans la littérature également, l'usage systématique du préservatif par les jeunes est moins fréquent [6, 15-17]. Cette faible utilisation du préservatif est liée en partie au fait que ces jeunes font le plus souvent des rapports sexuels occasionnels à un endroit où le préservatif n'est pas accessible et ne voulant pas perdre l'occasion, ils ont des rapports sans se protéger. La faible disponibilité du condom a été également évoquée comme cause de sa faible utilisation par Atuyambe *et al.* en Uganda en 2013 [15] et Gupta *et al.* dans six pays Africains [6]. Par contre, Potoczny *et al.* rapportent que l'utilisation du préservatif est influencée par le type de pratiques sexuelles et il est utilisé dans 40% des cas lors de la pénétration vaginale, 50% lors de la pénétration anale contre 3% lors des pratiques uro-génitales [18]. Isiugo-Abanihe *et al.* ont rapporté que l'utilisation du préservatif est assez élevée, surtout quand il s'agit des relations sexuelles impliquant des partenaires non réguliers [19]. La fréquence d'utilisation des méthodes contraceptives rapportée dans cette étude (35,4%) est inférieure à celles trouvées par Pettifor *et al.* (50% pour toutes les méthodes) [7] et par Godeau *et al.* (76,3% pour les préservatifs, 34,4% pour la pilule) [8]. Le multi partenariat a également été constaté par Mabiala Babela à Brazzaville avec 81,3% des garçons et 51,1% des filles qui ont déclaré avoir des multiples partenaires sexuels [1].

Au nombre des élèves adolescents ayant commencé les rapports sexuels, il y a 23,5% qui ont contracté une IST, 7,7% le VIH et 31,2% une grossesse. Près de la moitié de ces grossesses s'est soldée par un avortement soit provoqué clandestin (30,8%) ou spontané (17,3%). Les filles et les garçons (p=0,347), les écoles publiques et privées (p = 0,454) sont touchés de la même façon par ce phénomène. Par contre, les sorties nocturnes (p = 0,000), l'usage du tabac (p = 0,000), l'usage de l'alcool (p = 0,000) et l'usage de la drogue (p=0,000) apparaissent comme des facteurs associés à la réalisation des rapports sexuels chez les élèves. Ce même constat a été fait par Godeau *et al.* qui ont trouvé une association significative entre les rapports sexuels précoces avant l'âge de 15 ans et l'usage du tabac, de l'alcool, de la drogue et les sorties nocturnes [8]. Après avoir pris en compte l'interaction entre ces différents facteurs associés à la réalisation des rapports sexuels, seules les sorties nocturnes ont un lien significatif avec la pratique de relations sexuelles.

## Conclusion

Les rapports sexuels sont fréquents chez les élèves adolescents de la commune de Matoto et touchent pratiquement un élève adolescent sur deux. Dans la majorité des cas, il s'agit des rapports sexuels consentis avec une faible utilisation du préservatif et des autres méthodes contraceptives. Les facteurs qui sont significativement associés à la pratique des rapports sexuels sont les sorties nocturnes. Face à cette situation, il doit être envisagé une sensibilisation des élèves sur les dangers liés aux rapports sexuels et la nécessité d'utiliser le préservatif et les autres méthodes contraceptives.

### Etat des connaissances actuelles sur le sujet

- L'adolescence coïncide au début des rapports sexuels chez plusieurs personnes;
- La Guinée a un des taux de rapports sexuels chez les adolescents tout sexe confondus les plus élevés en Afrique;
- Les rapports sexuels à cet âge entrainent un risque accru d'infection par les IST/VIH, de grossesses non désirées, d'avortements à risque et d'abandon scolaire.

### Contribution de notre étude à la connaissance

- Ressortir la fréquence élevée du multi partenariat sexuel chez les élèves de la plus grande commune de Guinée;
- Identifier les facteurs qui favorisent la pratique de ces rapports sexuels;
- Ressortir la prévalence des infections sexuellement transmissibles y compris le VIH, des grossesses non désirées et des avortements clandestins chez cette catégorie d'adolescentes.

## Conflits d’intérêts

Les auteurs ne déclarent aucun conflit d'intérêts.
